# Ten Simple Rules for Getting Help from Online Scientific Communities

**DOI:** 10.1371/journal.pcbi.1002202

**Published:** 2011-09-29

**Authors:** Giovanni M. Dall'Olio, Jacopo Marino, Michael Schubert, Kevin L. Keys, Melanie I. Stefan, Colin S. Gillespie, Pierre Poulain, Khader Shameer, Robert Sugar, Brandon M. Invergo, Lars J. Jensen, Jaume Bertranpetit, Hafid Laayouni

**Affiliations:** 1 Institute of Evolutionary Biology (UPF-CSIC), Departament de Ciències Experimentals i de la Salut, Barcelona, Spain; 2 Institute of Organic Chemistry Universität Zurich, Zurich, Switzerland; 3 EMBL-EBI, Wellcome-Trust Genome Campus, Hinxton, United Kingdom; 4 California Institute of Technology, Biology Division, Pasadena, California, United States of America; 5 School of Mathematics & Statistics, Newcastle University, Newcastle upon Tyne, United Kingdom; 6 DSIMB, INSERM, U665, Paris, France; 7 Univ Paris Diderot, Sorbonne Paris Cité, UMR-S665, Paris, France; 8 Institut National de la Transfusion Sanguine, Paris, France; 9 Division of Cardiovascular Diseases, Mayo Clinic, Rochester, Minnesota, United States of America; 10 National Centre for Biological Sciences (TIFR), GKVK Campus, Bangalore, India; 11 NNF Center for Protein Research, Faculty of Health Sciences, University of Copenhagen, Copenhagen, Denmark; University of California San Diego, United States of America

## Introduction

The increasing complexity of research requires scientists to work at the intersection of multiple fields and to face problems for which their formal education has not prepared them. For example, biologists with no or little background in programming are now often using complex scripts to handle the results from their experiments; vice versa, programmers wishing to enter the world of bioinformatics must know about biochemistry, genetics, and other fields.

In this context, communication tools such as mailing lists, web forums, and online communities acquire increasing importance. These tools permit scientists to quickly contact people skilled in a specialized field. A question posed properly to the right online scientific community can help in solving difficult problems, often faster than screening literature or writing to publication authors. The growth of active online scientific communities, such as those listed in Table S1, demonstrates how these tools are becoming an important source of support for an increasing number of researchers.

Nevertheless, making proper use of these resources is not easy. Adhering to the social norms of World Wide Web communication—loosely termed “netiquette”—is both important and non-trivial.

In this article, we take inspiration from our experience on Internet-shared scientific knowledge, and from similar documents such as “Asking the Questions the Smart Way” [1] and “Getting Answers” [2], to provide guidelines and suggestions on how to use online communities to solve scientific problems.

## Rule 1. Do Not Be Afraid to Ask a Question

Some people are afraid of asking a question in public, for fear of appearing ignorant or foolish. Other people worry about their ability to express the question proficiently or with the correct grammar.

Actually, asking a question in a public website is a good thing. First, the process of composing a message to explain a problem is itself a great exercise. Second, it is a great way to learn faster, and to enter into contact with people from different fields. Third, and more importantly, your career will be difficult if you do not learn how to get help from other people.

As Albert Einstein once said, “The important thing is not to stop questioning. Curiosity has its own reason for existing” [3]. Asking the right questions should always be a priority in science, and online communities are a good place to practice.

## Rule 2. State the Question Clearly

The key to getting a good answer is to ask the question in a clear and concise way. If your question is too long, many people simply will not read it. On the contrary, if your question is too short, people may interpret it incorrectly and give you an erroneous answer.

A way to keep your questions short and concise is to systematically break down the problem into smaller parts. This can help you to decide where to seek help, and how much to seek. If you feel your problem is composed of multiple questions, then post as many messages as needed. You should start a separate discussion thread for each of the problems you want to solve, avoiding mixing messages about different topics together.

On the other hand, you should provide enough details so that people can answer you without having to ask you for additional explanations. Read the message you wrote carefully, and think about which details you forgot to include. A reader should be able to answer you just by reading your initial message, without having to look at the rest of the discussion, or at what other people already have said in response.

Some examples of non-concise questions and how to improve them are shown in Text S1. Spend as much time as you need in preparing your initial message: this will save time later and will lead you to find the best solution more easily. Many people are surprised to see how sometimes, in thinking about how to pose the problem, the answer reveals itself!

## Rule 3. New to a Mailing List? Learn the Established Customs before Posting

A common error is to rush into a web forum and start asking something without understanding how its web interface works and which people use the resource. Instead, a good habit is to spend a few days, after having created an account, reading the discussions published and practicing with the web interface. You will see which people use the forum or mailing list, which rules of netiquette are used, which kind of questions are asked, and how much time it takes to obtain an answer. For this reason, it is a good idea to subscribe to a few mailing lists or forums on your topics of interest even when you do not urgently require anything from them. This will show you the concrete ways in which people post messages.

Remember that you may have to use a different language depending on the audience you are addressing. For example, some technical terms may be understood in one mailing list or community but not in others. People who do not study genomics might not immediately know how to respond to questions about GWASs, SNPs, or STRs (genome-wide association studies, single nucleotide polymorphisms, and single tandem repeats, respectively).

## Rule 4. Do Not Ask What Has Already Been Answered

People in general do not like to repeat their explanations. Before posting a question, use a search engine to see if a similar question has been asked previously. You should post a new question only if the answers you have found are not satisfactory. In case you decide to post a new question, cite the previous answers and explain why they are not sufficient to solve your problem. This demonstrates that you have already researched the answer on your own. Most discussion forums or mailing lists also have a searchable archive, which should be consulted before posting a question.

## Rule 5. Always Use a Good Title

People like to quickly skim through titles, looking for questions within their expertise that they are able to answer. So, you will have to be good at catching the attention of the readers that can help you. Use a clear and concise title, so that readers can decide whether they are able to respond to your message without having to read the whole message.

An approach to choosing a good title is to think of a hypothetical web search query that you would use to find a solution to your problem. For example, where you might search for “format BLAST database,” an adequate title for a forum post could be “How do I format a BLAST database?” or “Formatting a BLAST database.” More specificity, within reason, is preferable.

At the same time, it is important not to waste the time of the people who are not able to help you, and are not interested in what you are writing. Refrain from attempts to attract attention with titles such as “Help me” or “Urgent.” People usually do not appreciate these kinds of titles because each forum member must then view the post in order to understand what you are asking. If you use incorrect titles, your message may be censured or closed by the moderators, and you may be forbidden to use the resource.

Some examples of good and bad titles are shown in Text S1.

## Rule 6. Do Your Homework before Posting

People in an online community are willing to help, but are not there to work for you. You should always show that you have first tried to solve your problem by yourself. Explain clearly what you have done, and describe the approach that you took.

When asking for help to solve an assignment, always explain how you have tried to solve it. Many students from bachelor programs use web forums and mailing lists to copy-paste the assignments given by their teachers, and call on other people to show them how to solve them. This behavior is not well received and can bring you a bad reputation.

However, you can nonetheless ask for help on how to solve an exercise if you demonstrate that you have made some effort in solving it. Show what you have done so far, and why you think it is not correct. Ask other people to check your solution, not to give the solution to you.

When asking about a programming issue, do not expect other people to write a whole program for you: rather, post an example of the code that you have written and where you are stuck. Include an example of the input and the expected output of your program. If you receive error messages, also include the full output of the error. This will help the other users to inspect your logic, to test the code on their own computers, and to easily pinpoint the problem therein.

If you ask a question about a software package, make sure that the solution is not already answered in the user manual or the Frequently Asked Questions (FAQs) before bringing your question to a forum. Also, declare that you have already checked these sources.

If you really need another person to write a program or a task for you, then explain that you are looking for a collaboration, and say how you will acknowledge a correct answer. If you explain everything well, your reputation online will also improve.

## Rule 7. Proofread your Post and Write in Correct English

Using correct grammar is important. Readers will be more likely to answer if the question is clear and correctly posed. Your grammar does not need to be academic, but it must be intelligible to a broad audience. Avoid slang and abbreviations as much as possible, to show that you have made at least some effort in writing a clear message. Writing in capital letters or in unconventional styles, such as that of text messages, is usually unwelcome, and in the long term can deteriorate your reputation online.

Your message should be as concise as possible. You do not need to introduce yourself on every message; doing it only once will be enough. Be careful of using too many adverbs and adjectives, or unnecessary changes in verb tense, as they may make the text difficult to understand. Also, do not be afraid of repeating technical terms more than once, as using too many synonyms will only make the text more difficult to understand.

This rule may be the most difficult to follow for non-native English speakers. A good approach is to spend some time reading the messages written by other users of the forum or the mailing list and follow their example. Search for a question similar to what you want to ask, and use it as a model; you may even copy and paste some portions of the text if it helps you to formulate a correct question.

## Rule 8. Be Courteous to Other Forum Members

Members of a discussion forum are usually unpaid volunteers who offer their time and expertise by volition and not by obligation. They are therefore not obliged to answer any questions at all.

Maintaining civil and polite conversations fosters an environment that encourages people to contribute. You must remember that forums are as human as their users, and you may sometimes receive a perfect answer written in an unfriendly tone. This can happen for various reasons: perhaps the same question was asked previously, or maybe the author was in a bad mood when writing. For your career, it is crucial that you not permit the discussion to degenerate into an argument. Even if you receive an impolite answer, stay calm and answer as gently as you can [4]. And remember the golden rule: treat other forum members as you wish to be treated.

One of the most impolite behaviors toward an online community is asking a question in multiple places at the same time. “Cross-posting”, as this practice is called, can make two distinct online communities work through a solution for you when only one is needed; this is an abuse of forum members' time. If you have not received an answer and you believe that asking it in another place would get you one, provide a link back to the original discussion. Similarly, if you receive an answer in a different forum, report the answer to the original forum. Then, the people who helped you will know what the correct solution is and that you are no longer looking for it.

## Rule 9. Remember That the Archive of Your Discussion Can Be Useful to Other People

Messages in a mailing list or forum remain archived on the Internet. In certain situations, this can be a source of trouble: check the policy of your university or employer regarding posting on the Internet; avoid spreading embargoed information; and if possible, use your academic/corporate email address when registering, to keep your private life separated from your work.

Nevertheless, most of the time it is possible to make use of online communities without breaking any of your employer's rules. In these cases, the fact that an archive of the discussion remains publicly accessible is positive, as it becomes a useful resource for people searching for solutions to similar problems. Several knowledge archives are actively saving bioinformatics-related questions from open source projects. For example, questions about BioPerl [5] are kept in the GMANE (http://news.gmane.org/gmane.comp.lang.perl.bio.general) and Nabble archives (http://old.nabble.com/BioPerl-f13596.html).

Since an archive of the discussions remains available on Internet, it is good practice to conclude the discussion by indicating the correct solution to the problem exposed or by summarizing the suggestions received. If some of the answers that you received have proven to be wrong, do not be afraid of writing it in the online discussion: this will help other people avoid trying an erroneous solution. Even if you did not receive any useful answers, sacrifice a bit of your time to thank the people who tried to help you and to explain that you were not able to find a solution.

## Rule 10. Give Back to the Community

Have you found your answer? Great! As time progresses and you get more experienced in the respective field in which you asked your question, you might want to start contributing the knowledge that you have gained by helping people that are now in your previous position. Most online communities are very welcoming to new members, as they alleviate the work of more experienced ones. Also, as a new contributor, you might be able to see problems from a beginner's point of view. You do not have to contribute to the community by answering questions, as some communities have a “wiki-style” interface where you can contribute by editing, tagging, or flagging questions. In any case, following at least a few science-related mailing lists and contributing actively to them is a great way to come into contact with researchers working in your field, and over time can lead you to new collaborations and new opportunities for your career.

## Supporting Information

Table S1 List of bioinformatics- and biology-related mailing lists and communities.
(DOC)

Text S1 Examples of poorly posed questions, and how to improve them.
(DOC)
