# Supplementary material for: Ten Simple Rules for Getting Help from Online Scientific Communities
Source: PLoS Comput Biol. 2011 Sep 29;7(9):e1002202. doi: 10.1371/journal.pcbi.1002202 (PMC3182872; doi:10.1371/journal.pcbi.1002202)
Supplement: Table S1 — List of bioinformatics- and biology-related mailing lists and communities. (DOC) [file pcbi.1002202.s001.doc]

| **Name** | **Since** | **Approximate number**  **of messages** | **Description** | **URL** |
| --- | --- | --- | --- | --- |
| BioBB | 2001 | 6,300 | Bio bulletin board mailing list (maintained by the Bioinformatics.org community) | http://www.bioinformatics.org/pipermail/bbb/ |
| BioC | 2001 | 46,200 | The bioconductor Project Mailing list | https://stat.ethz.ch/mailman/listinfo/bioconductor |
| BioPerl | 1996 | 44,700 | The BioPerl project (users and developers mailing lists) | http://www.bioperl.org/wiki/Mailing_lists |
| BioPython | 1999 | 7,300 | The BioPython project (users and developers mailing lists) | http://lists.open-bio.org/pipermail/biopython/ |
| BioRuby | 2004 | 2,000 | The BioRuby project (users and developers mailing lists) | http://lists.open-bio.org/pipermail/bioruby/ |
| BioStar | 2009 | 14,300 | Questions and answers on bioinformatics, computational genomics and systems biology | biostar.stackexchange.com |
| Blue Obelisk | 2009 | 500 | Use and development of Open Data, Open Source, and Open Standards for chemistry tool, service, or database | blueobelisk.shapado.com |
| CCP4 bulletin board | 2007 | 21,100 | CCP4 suite, and macromolecular crystallography in general | https://www.jiscmail.ac.uk/cgi-bin/webadmin?A0=CCP4BB |
| gmx-users | 2000 | 72,200 | GROMACS molecular dynamics software | http://lists.gromacs.org/pipermail/gmx-users/ |
| MetaOptimize | 2010 | 2,120 | Community of scientists interested in machine learning, natural language processing, artificial intelligence, text analysis, information retrieval, search, data mining, statistical modeling, and data visualization, as well as adjacent topics | http://metaoptimize.com/qa/ |
| Molecular Station | 2003 | 410,500 | One of the most complete and supported bio-forum on the internet | http://www.molecularstation.com/ |
| Protocol Online | 1999 | 104,000 | Life sciences lab protocols supported by a community | www.protocol-online.org |
| R-help | 1997 | 307,700 | The R programming language | https://stat.ethz.ch/mailman/listinfo/r-help |
| Stackoverflow | 2008 | 10,000,000 | Q&A for professional and enthusiast programmers | stackoverflow.com |
| Stats | 2010 | 21,200 | Q&A for statisticians, data analysts, data miners and data visualization expert | stats.stackexchange.com |
