# Supplementary material for: Ten Simple Rules for Getting Help from Online Scientific Communities
Source: PLoS Comput Biol. 2011 Sep 29;7(9):e1002202. doi: 10.1371/journal.pcbi.1002202 (PMC3182872; doi:10.1371/journal.pcbi.1002202)
Supplement: Text S1 — Examples of poorly posed questions, and how to improve them. (DOC) [file pcbi.1002202.s002.doc]

|  |
| --- |

# S[upplementary table 2 for 'Ten Simple Rules': examples of p](http://www.wikigenes.org/e/art/e/144.html?vs=33)oorly posed questions, and how to improve them

1. Example 1: question that can be splitted into multiple questions.

| **Original Question** | **Improved Question**  Split the question!  If you have more  than one question  to ask, split it  Into multiple  discussions.  (see rule #2) |
| --- | --- |
| **biopython**    Hello people,  I am trying to install biopython on a Ubuntu 11.04 64-bit machine. I followed the documentation on the wiki, but I don't understand whether I should be using the easy_install egg or download from github.  Always ask only  one single question  for each message.  Asking too many  questions is  confusing, and  people do not  know what to  answer first.  (see rule #2)  Moreover, I need to write a script to parse Fasta files using biopython. Can anyone explain me how to do it?  Finally, I would like to know if biopython can also read fastq files. | **Installing biopython on Ubuntu**  Hello people,  I need to install biopython on a Ubuntu 11.04 machine.  I followed the documentation on the wiki, but I don't understand whether it is better to use the easy_install egg or to download from github.  What do you recommend me? |
|  | **Parsing Fasta sequences with biopython**  I need to write a script to parse Fasta files using biopython. So far, I wrote the following code (..) |
|  | **Can biopython read fastq files?**  Hello,  does anyone know how can I read fastq files with biopython? |

1. Example 2: question having a poor subject and missing information.

Avoid titles like

'Help!', 'Urgent!',

'Help needed!'.

(see rule #5)

| **Original Question**  If the title is good,  people are able to  understand what  the question is  about before reading  your message.  (see rule #5) | **Improved Question** |
| --- | --- |
| **Help needed urgently...!!!**    Dear members,  My name is Frank from the University of …. For my thesis project, I am working on simulation of certain models of protein myosin. We are interested in predicting a three dimensional structure of this protein in mouse.  Be concise. You  don't need to  Explain your full  background  everytime.  (see rule #2)  When I use the grompp_mpi (for position restraint), it gives me an error message as "Fatal error: Invalid line in em.gro for atom 3642:" and the program is aborted.  Check if you are  providing all the  details needed to  answer you.  Which version of  the software are  you using?  Have you looked at  the documentation?  (see rule #2)  Be sure that  you are providing  all the details  needed to  answer you,  such as software  version,  the command you  executed,  and so on.,  (see rule #2)  Could you please tell me what could be the possible mistake??  Waiting for your reply...  Thanks | **Gromacs: fatal error when parsing file**  Dear members,  I am having problems parsing files with gromacs.  This is the command line that I am executing:  $: grompp_mpi -np 32 -f em_cc.mdp -po det_em2.mdp -c em.gro -p myprot_em.top -o myprot_em.tpr  When I call this command line, it gives me the error:  “Fatal error: Invalid line in em.gro for atom 3642:”  I searched the manual for this error, but did not find any references. Can you see where the error is?  I am running gromacs 4.5.1 on a Linux 64 bit machine and Ubuntu 11.04 as operating system. |

1. Example 3: question where the user asks for help to solve an assignment.

| **Original Question** | **Improved Question** |
| --- | --- |
| **Double stranded DNA**    Hey guys, here I report one of the exercises I had yesterday in the examination of Organic Chemistry:    "How can you determine quickly, whether a particular DNA forms double strands or not by NMR?"  Other people  will not solve  assignments or  do homework  for you.  (see rule #6)  Who knows the answer? Thanks! | **The use of NMR for determining the contacts in a DNA double helix.**    Dear all,  I am trying to solve a question from an exam of Organic Chemistry I had yesterday.  The problem says:  "How can you determine quickly, whether a particular DNA forms double strands or not by NMR?".  My reasoning is that in a double-stranded DNA the imino protons are hydrogen-bonded, so exchange with water is slow and the lines are comparably sharp. So, NOE (Nuclear Overhauser Effect ) information could be used to discern double-helix DNA strands by NMR.  Explain how you  tried to solve the  problem,  and ask people to  check it.  (see rule #6)  Can you please tell me if my reasoning is correct? |

1. Example 4: questions where the user asks other people to write a software.

| **Original Question**  Demonstrate  that you have  tried to solve  the problem  by yourself  (see rule #6) | **Improved Question** |
| --- | --- |
| **Software to parse fasta sequences**    Other people will  not your work  for you.  (see rule #6)  Hello,  I need to write a software to parse a fasta sequence. Can you please write it for me? | **Software to parse fasta sequences**  Hello,  I need to write a software to parse a fasta sequence.  So far, I wrote the following python code:  filename = 'myseq.fasta'  sequence = ''  for line in open(filename, 'r'):  if not line.startswith('>'):  sequence += line  print sequence  How can I improve this code? Do you see any error?  Thanks |
|  | **Collaboration needed – software to parse fasta sequences**  Alternatively,  if you need other  people to do  something for you,  explain  how you will  acknowledge them  (see rule #6)  Hello people,  my group needs a software to parse fasta sequences.  If anyone can help us and write the software for us, we will acknowledge it in the paper we are writing.  Here are some more information (….) |

1. Example 5: incomplete question.

Use an informative

title
(see rule #6)

| **Original Question** | **Improved Question** |
| --- | --- |
| **Imidazole concentration**    Dear all,  what is the imidazole concentration for the washing step?  This question is  not complete:  people will have to  ask for more details  to understand what  the question  is about.  (see rule #2)  Provide all the details  needed to understand  what are you doing  and what is the  problem you are  facing.  (see rule #2) | **Imidazole concentration in the washing step on a nickel column.**  Dear all, I am experiencing troubles with the purification of my protein from *E.coli*.  I am following the protocol in (references). During the washing step with 50mM imidazole I loose quite a lot of protein. I know that this concentration of imidazole is high, but if I use less I have more contaminants in my elution.  What concentration for imidazole do you recommend me? Can you point me to some literature reference on what is the best concentration for imidazole?  Thank you very much. |

1. Example 6: question written in not-concise English.

| **Original Question** | **Improved Question** |
| --- | --- |
| **How to calculate the micelle size of a detergent?**  Hello,  Be concise.  Avoid using too  many adverbs  and adjectivs.  Also, do not abuse  of transitions like  'These days',  'In fact', 'eventually',  and others  (see rule #7)  Prefer simple  sentences, go  straight to the  point.  (see rule #7)  These days I have been looking for a method that would allow me to calculate how big (in kDa) are the micelles formed by the detergent DDM.  I am wondering whether somebody could explain me which formula I could use to calculate the micelle size. In fact, even after looking in pubmed, I haven't found anything useful.  I will be very grateful if you can help me solving this problem. | **How to calculate the micelle size of a detergent?**  Hello,  I am looking for a method to calculate the size of the micelles formed by the detergent DDM.  Does anyone know which formula I can use? I have searched on pubmed, but could not find any reference.    Thanks in advance. |
